# Quinolizidine Alkaloid Profiles in Lupin-Based Products: Monitoring of the Italian Retail Market and Efficacy of the Debittering Process

**DOI:** 10.3390/foods15132269

**Published:** 2026-06-24

**Authors:** Mariantonietta Peloso, Ilaria Prizio, Gaetan Minkoumba Sonfack, Eleonora Baraldini Molgora, Elisabetta Caprai

**Affiliations:** National Reference Laboratory for Plant Toxins in Food, Food Chemical Department, Istituto Zooprofilattico Sperimentale della Lombardia e dell’Emilia-Romagna “Bruno Ubertini” (IZSLER), Via P. Fiorini 5, 40127 Bologna, Italy; m.peloso@izsler.it (M.P.); ilaria.prizio@izsler.it (I.P.); g.minkoumbasonfack@izsler.it (G.M.S.); e.baraldinimolgora@izsler.it (E.B.M.)

**Keywords:** quinolizidine alkaloids, *Lupinus*, LC-MS/MS, plant-based products, debittering

## Abstract

The increasing interest in plant-based diets has driven the demand for sustainable legumes such as lupins. However, their broader utilization is limited by the presence of quinolizidine alkaloids (QAs), secondary metabolites toxic to human health. Despite severe health risks, European legislation lacks harmonized maximum levels, necessitating comprehensive market surveillance. This study evaluates the occurrence of 14 specific QAs across 28 commercial samples collected from the Italian market using a validated LC-MS/MS method. Concurrently, the impact of a laboratory-scale debittering process was examined on a subset of eight dried seed samples to monitor toxin reduction efficiency. The total alkaloid content exhibited significant variability across food categories. Dried lupins samples showed the highest concentration (mean: 13,676 mg/kg), whereas lower levels were detected in lupin flours (mean: 253 mg/kg), brined lupins (mean: 86.1 mg/kg), and ready-to-use products (mean: 70.3 mg/kg), with lupanine being the predominant alkaloid across most matrices. Data on finished lupin-based products confirm the efficacy of industrial processing techniques in significantly reducing the alkaloid content. In parallel, laboratory debittering process demonstrated high QA reduction efficiency, ranging between 94% and 99%. Nevertheless, if initial QA levels were high, most of the debittered lupin seeds still retained residual alkaloid levels exceeding commonly accepted safety thresholds. This highlights the importance of establishing harmonized regulatory limits within the European Union and implementing continuous analytical monitoring programs, both at the field level and on commercial products, to guarantee consumer safety.

## 1. Introduction

The increasing adoption of plant-based diets represents a strategic response to the environmental challenges posed by livestock farming, aiming to mitigate greenhouse gas emissions and safeguard biodiversity [1]. In this context, lupins have emerged as a sustainable alternative to soybean, whose intensive cultivation is a primary driver of global deforestation [1,2]. Beyond their low environmental impact, these legumes play a fundamental ecological role: they are capable of fixing atmospheric nitrogen into the soil, which is why they have historically been utilized in crop rotation to counteract erosion and structurally improve soil quality [3]. Characterized by a robust nutritional profile with protein and fiber content each reaching approximately 40% [4,5,6], these legumes are increasingly utilized as functional ingredients in meat analogs, bakery products, beverages, and dairy alternatives [7,8]. However, expanding the food valorization of the *Lupinus* genus requires addressing safety challenges posed by the presence of quinolizidine alkaloids (QAs). These lysine-derived secondary metabolites are synthesized in the green tissues as a natural defense mechanism, subsequently accumulating in all plant organs, including the seeds [8]. While QAs have predominantly been isolated from the Fabaceae family, it is worth noting that they are also present in other plant families [8,9]. Within this genus, over 500 lupin species have been described, but only 12 are widespread in Europe. Worldwide, only these four species are cultivated on a commercial scale for both human consumption and animal feed: white lupin (*L. albus*), blue lupin (*L. angustifolius*), yellow lupin (*L. luteus*), and Andean lupin (*L. mutabilis*) [8]. The concentration of QAs in these plants depends on a complex interaction between genotype and environmental factors. A sharp distinction exists between “sweet” cultivars, bred for minimal alkaloid content (less than 500 mg/kg total QAs), and “bitter” varieties, which may harbor high concentrations (more than 10,000 mg/kg total QAs) [8,10].

The presence of QAs represents a significant food safety concern for consumers of lupin and lupin-related products, as these metabolites interfere with nicotinic and muscarinic cholinergic receptors, exerting multi-systemic toxicity that affects the nervous, circulatory, and digestive systems [2]. Clinical manifestations of poisoning include dizziness, cognitive disorientation, and tachycardia, often accompanied by gastrointestinal distress such as nausea and emesis. Common anticholinergic effects, including xerostomia and ataxia, are frequently observed; in cases of severe intoxication, the condition may progress to cardiac arrest or lethal respiratory paralysis [11,12]. At the same time, lupin proteins pose a severe immunological hazard; lupin allergens can trigger acute primary or cross-reactive allergic reactions, often in patients with pre-existing peanut or soy allergies, ranging from hives to anaphylactic shock [13].

To ensure the safety of these legumes, rigorous debittering, consisting of sequential soaking and boiling, is necessary to leach the toxins, a process capable of eliminating up to 97% of the total QA load [8]. Nevertheless, recent evidence suggests that even sweet varieties may exhibit elevated alkaloid levels due to climatic stressors [10].

Indeed, QA biosynthesis and accumulation are highly sensitive to environmental factors, including light, elevated temperatures, and drought stress, dynamics that have become increasingly critical due to climate change. For instance, drought conditions during early plant development can significantly elevate QA levels. The synthesis of these compounds accelerates under combined environmental and biotic stressors, serving as a chemical defense mechanism for the plant [3].

From an agronomic and safety perspective, these climate-driven fluctuations pose a constant challenge to maintaining low-alkaloid traits. This vulnerability is further complicated during seed propagation because the low-alkaloid character is a recessive trait. Consequently, crops face a continuous risk of genetic drift driven by cross-pollination from bitter or wild populations, which progressively intensifies across successive generations due to natural selection pressures [3].

Despite these hazards, regulatory oversight remains globally inconsistent: while the Food Standards Australia New Zealand (FSANZ) enforces a limit of 200 mg/kg for lupin derivatives and 5 mg/kg for sparteine in alcoholic beverages [14], the European Union lacks harmonized legal limits. Historically, the UK Advisory Committee on Novel Foods and Processes (ACNFP) first recommended a 200 mg/kg QA limit for *L. angustifolius* in 1996, followed by France in 1998, which restricted *L. albus* flour to a 10% maximum under the same 200 mg/kg threshold [13,15,16].

In 2019, the European Food Safety Authority (EFSA) CONTAM Panel established, for acute exposure, a lowest relevant single oral effective dose of 0.16 mg sparteine/kg body weight as the toxicological reference point. This value is derived from the lowest therapeutic dose of sparteine sulfate used in treating cardiac arrhythmia, where anticholinergic effects represent the primary adverse clinical outcome. Consequently, EFSA adopted a group approach based on dose additivity, necessitated by the similar modes of action of QAs. Conversely, no reference point could be identified for chronic exposure due to insufficient toxicity data [8].

More recently, in 2024, the European Commission has issued a monitoring recommendation for Member States to actively collect data on QAs in lupins and derived products [17]. In line with this regulatory direction, the framework has been further updated by the Commission Recommendation (EU) 2026/1241 of 11 June 2026, which reinforces the mandate for continuous analytical monitoring across the European Union [18].

Given the absence of harmonized legislation alongside the rapid market expansion of lupin-based products, establishing comprehensive analytical surveillance is essential to guarantee consumer safety.

Historically, the food use of whole lupin seeds was restricted to local dietary habits in the Mediterranean area, primarily as a snack or accompanying traditional dishes in Southern Italy [19]. However, data from the statistical database of Food and Agriculture Organization (FAOSTAT) reveal a significant surge in the Italian lupin industry, with production volumes more than doubling from 810 tonnes in 2019 to 1740 tonnes in 2024 [20].

This rapid transition from a niche traditional food to a widely distributed industrial ingredient underscores the critical importance of monitoring alkaloid levels.

To date, substantial data gaps remain regarding both the concentration of QAs in lupin-based products and the actual consumption patterns within the general population. Furthermore, comprehensive toxicity data for individual QAs are still lacking [8,13].

To address this evolving scenario, the present study evaluates 14 specific QAs across 28 samples collected on the Italian market using an LC-MS/MS validated method. This study aims to provide a more detailed characterization of the total alkaloid profile in commercial products. Furthermore, the research examines the influence of household processing, including boiling, soaking, and dehulling, on toxin reduction, seeking to confirm the efficacy of debittering protocols and establish updated exposure data for these compounds.

## 2. Materials and Methods

### 2.1. Sampling

A total of 28 commercial lupin-based samples were collected on the Italian market between 2025 and 2026 as part of the official Italian Monitoring Plan for Plant Toxins.

To ensure a comprehensive and independent sampling design, each sample within a specific category represents a distinct production batch and a different commercial brand.

Specifically, the dried lupin packaging explicitly stated that the seeds were raw and featured instructions for domestic debittering (soaking and boiling) prior to consumption. The lupin flours were labeled as 100% obtained from milled, dehulled, and toasted sweet lupin seeds. Brined lupins contained only lupins and salt, commercialized either vacuum-packed or immersed in saltwater. Lastly, the ready-to-use products consisted of a vegetable salami formulation containing 26% lupin flour, and a toasted, salted, and pre-debittered lupin snack.

The sample categories and their distribution are summarized in Table 1.

### 2.2. Materials and Reagents

A total of 14 QAs, including the indole alkaloid gramine, were investigated in this study. Analytical standards were obtained from the following sources: albine (as hydrochloride), anagyrine (as hydrochloride), cytisine, 13α-hydroxylupanine, lupanine (as hydrochloride), methylcytisine, multiflorine, sparteine, and gramine were purchased from Phytolab (Vestenbergsgreuth, Germany); lupinine from Sigma-Aldrich (St. Louis, MO, USA); trans-13α-cinnamoyloxylupanine from Biosynth (Marseille, France); thermopsine from Cayman Chemical (Ann Arbor, MI, USA); and angustifoline and epilupinine (as hydrochloride) from TRC (Toronto, ON, Canada). LC-MS-grade methanol was purchased from VWR Chemicals (Rosny-sous-Bois-cedex, France), formic acid from Carlo Erba Reagents (Milan, Italy), and analytical-grade ammonium formate from Sigma-Aldrich (St. Louis, MO, USA). Ultrapure water was obtained from an EvoQua Water Technologies system (Diessechem, Milan, Italy).

### 2.3. Working Solutions

Stock standard solutions for each quinolizidine alkaloid (1000 µg/mL) and subsequent working standard solutions containing a mixture of QAs (5 µg/mL and 500 ng/mL) were prepared in methanol. All solutions were stored at −20 °C.

### 2.4. Sample Preparation

A total of 28 samples were processed for analysis. Among these, 8 samples of dried lupins were specifically selected to undergo a laboratory-scale debittering process according to the label specifications.

#### 2.4.1. Extraction of Alkaloids from Lupin Products

For the extraction, a 2.0 ± 0.1 g aliquot of each homogenized sample was extracted with 40 mL of a methanol/water/formic acid mixture (50:50:0.1, *v*/*v*/*v*) and shaken for 30 min on an oscillating shaker. The extracts were then centrifuged at 3500 rpm for 15 min. An aliquot of the supernatant was diluted with water/methanol (90:10, *v*/*v*) and transferred to a 500 µL filter vial for analysis, following the EURL-Mycotoxins and Plant Toxins method_012 (v. 1) by Wageningen Food Safety Research [21].

To ensure that QA concentrations complied with the linear range of the calibration curve (detailed in the next section), seed extracts were further diluted 5-, 25-, or 100-fold.

#### 2.4.2. Debittering Process Simulation

To evaluate the reduction kinetics of quinolizidine alkaloids, a subset of eight dried lupin samples was subjected to a laboratory-scale debittering protocol.

**Figure 1:**
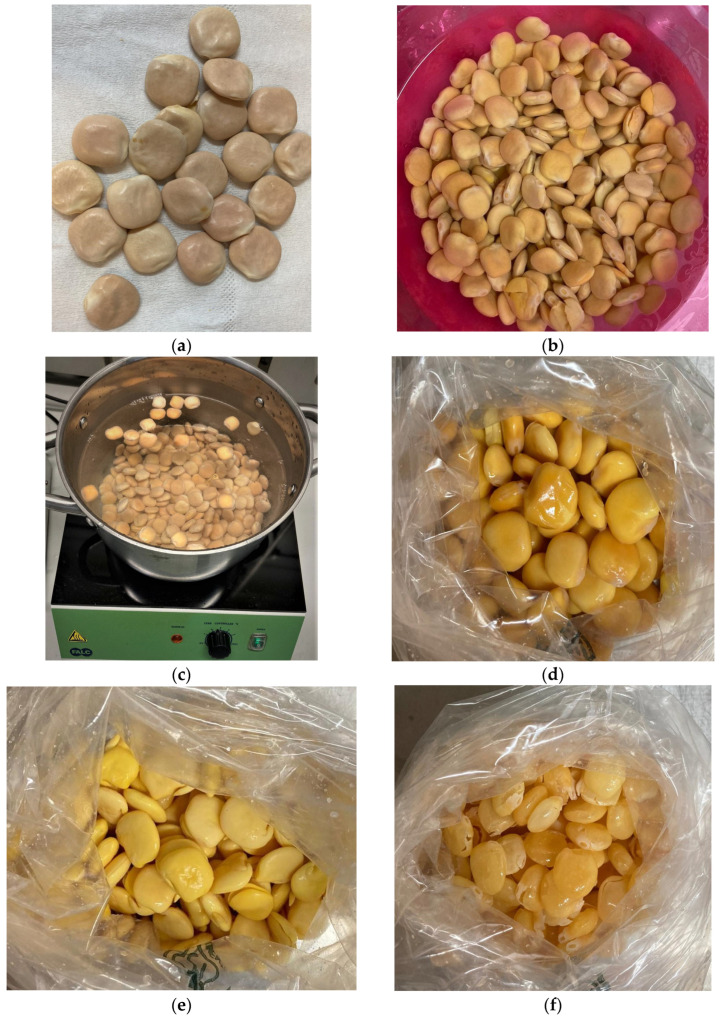
Debittering laboratory simulation: (**a**) dried lupin seeds; (**b**) soaking phase; (**c**) boiling phase; (**d**) the whole debittered lupin seeds; (**e**) the dehulled debittered lupin seeds; (**f**) lupin seed hulls (IZSLER photographs, 2025).

Despite the availability of diverse debittering methods in the literature, a standardized protocol remains lacking [8]. Therefore, the laboratory procedure was designed to simulate domestic processing by following the instructions provided on the product labels (Figure 1). The procedure involved soaking the dried seeds in cold water for 24 h. Subsequently, the seeds were boiled for 3 h using a 1:3 (*w*/*v*) sample-to-water ratio under gentle simmering conditions (approximately 95–100 °C, ensuring the hull softened uniformly. After boiling, the lupins were drained and immediately covered with fresh cold water. To remove the bitterness, the washing water was renewed three times a day at regular intervals over a continuous period of 3 days. Subsequently, the seeds were immersed in a brine solution (5% *w*/*v* NaCl) for final rehydration and preservation.

To systematically monitor QA reduction across the different processing stages, each of the eight dried lupin samples was divided into four distinct fractions, yielding a total of 32 individual sub-samples analyzed independently via LC-MS/MS. Specifically, this experimental design accounted for the following four sub-samples: (i) whole dried seeds (sampled before processing; Figure 1a); (ii) whole debittered seeds (sampled at the end of the entire protocol; Figure 1d); and (iii) dehulled debittered seeds (Figure 1e) along with (iv) their separated hulls (Figure 1f), both obtained by manually peeling the whole debittered seeds collected at the final stage. Prior to analysis, all sub-samples were ground and processed as detailed in the previous section (Section 2.4.1).

### 2.5. LC-MS/MS System and Chromatographic Conditions for Analysis

The LC-MS/MS system consisted of an Acquity UPLC I-Class PLUS chromatographic module (Waters Corporation, Milford, MA, USA) coupled with a quadrupole mass spectrometer Xevo^®^ TQ-XS (Waters Corporation, Milford, MA, USA), equipped with the managing software MassLynx V4.2 SCN1046 (Waters Corporation, Milford, MA, USA). The chromatographic separation was performed by using an Acquity UPLC BEH C18 column (130 Å, 1.7 μm, 2.1 mm × 100 mm) from Waters Corporation (Milford, MA, USA), heated at 50 °C. The detailed UPLC parameters, mobile phase gradient, and electrospray ionization (ESI+) source settings are summarized in Table S1. The mobile phase was delivered using a binary gradient: initial isocratic hold at 0% B for 1.0 min; gradient ramp to 5% B from 1.0 to 1.5 min; isocratic hold at 5% B from 1.5 to 2.5 min; gradient ramp to 60% B from 2.5 to 5.5 min; gradient ramp to 100% B from 5.5 to 7.0 min; followed by re-equilibration at 0% B for 1.0 min. The total run time was 8 min. The ideal ionization and fragmentation conditions for the analytes were identified using a continuous infusion of the tuning solution.

Target QAs were identified and quantified based on their retention times, specific mass transitions, and ion ratios (Table S2).

Quantification was carried out via external matrix-matched calibration curves prepared in a diluted blank soybean extract. Calibration standards were generated at concentrations of 0, 1, 2.5, 5, 10, 25, 50, 100, and 200 ng/mL. Calibration curves were constructed by plotting the peak area of each analyte against its nominal concentration.

### 2.6. Method Performance and Identification Criteria

The LC-MS/MS method was validated in house according to EURL-MP-guidance doc_003 (v. 1.4, 2024) and EURL-MP- method_0.12 (v.1, 2022) [21,22]. Quality control procedures included the assessment of linearity, accuracy (recovery rates), precision (repeatability and reproducibility), selectivity, and limit of quantification (LOQ) of the method.

According to SANTE/11312/2021, QAs were identified based on retention time, a target ion, and two qualifier ions [23]. Retention times were within ±0.1 min of the corresponding reference peaks, with peaks exhibiting similar shapes and co-elution. The ion ratios were within ±30% of the average values obtained from calibration standards in the same analytical sequence. All peaks were within the linear range of the detector and showed a signal-to-noise ratio (S/N) ≥ 3.

Chromatograms of matrix blanks showed no interfering peaks at the retention times of any QAs, confirming the high selectivity of the method; no blank lupin seeds were available, so soybeans were used as the surrogate blank material [22,24]. Linearity was observed over the concentration ranges of 1–200 mg/kg in lupin seeds and 0.2–40 mg/kg in food products. Samples with higher QA concentrations could be analyzed after dilution of the extract. The linear regression coefficient (R^2^) was ≥0.99 for all analytes in all matrices. The back-calculated concentrations of the calibration standards were ± ≤20% of their nominal values. Recovery values were within the acceptable range of 70–120%, while repeatability (RSD_r_) and within-laboratory reproducibility (RSD_R_) were below 20%, meeting the required precision criteria. The limit of quantification (LOQ) was 1 mg/kg for lupin seeds and 0.2 mg/kg for lupin-based products, ensuring sufficient sensitivity for QA detection in the analyzed matrices. Overall, the method can be considered fit for purpose for the determination of QAs in lupin seeds and food products.

## 3. Results and Discussion

### 3.1. Occurrence of QAs in Analyzed Samples

Total quinolizidine alkaloid content showed significant variability among the 28 analyzed samples, reflecting the diverse range of lupin-based products currently available on the Italian market (Table S3). As expected, the dried lupins category exhibited the highest levels, with a mean QA value of 13,676 mg/kg (range: 7716.0–18,662.6 mg/kg) (Table 2). These high concentrations confirm that raw, dried seeds contain substantial alkaloid loads, posing serious toxicological risks to consumers if not properly debittered. This aligns with recent European market surveys; for instance, Keuth et al. reported critically high concentrations reaching up to 21,000 mg/kg in unlabelled lupin seeds [7].

In contrast, levels in lupin flours were markedly lower (mean: 253 mg/kg), suggesting either the selection of “sweet” lupin varieties or efficient industrial debittering prior to milling. Brined lupins (mean: 86.1 mg/kg) and ready-to-use products (mean: 70.3 mg/kg) displayed the lowest concentrations, confirming that commercial washing and processing effectively reduce QAs to safe levels for human consumption, consistent with values reported for finished products in the EU [7,25]. Specifically, a survey of the German retail market monitoring five QAs across 30 lupin-based products showed that QA concentration in milling products and flours ranged between 113 and 609 mg/kg [7]. The same author evaluated processed lupin-based matrices, such as spreads, estimating the QA contribution of the lupin ingredient to be between 75 and 147 mg/kg. A similar trend of moderate, process-dependent QA retention was documented on the Belgian market by Schryvers et al. [25]. In their assessment of 7 QAs across processed lupin-based alternatives, mean concentrations reached 234 ± 135 mg/kg for lupin-based protein powders, 177 ± 195 mg/kg for egg alternatives, and 428 ± 368 mg/kg for coffee surrogates. The overall quantitative ranges from both Germany and Belgium support the data of the present study, confirming that standard European commercial processing effectively reduces the QA content.

Although the European Union has not established a harmonized maximum level (ML), the Australia New Zealand Food Standards Code stipulates a limit of 200 mg/kg for lupin flour, kernel flour, kernel meal, and hulls [14]. In the absence of specific EU thresholds, this limit is widely recognized as a global safety benchmark.

In our dataset, all ready-to-use products and most brined samples were compliant with this limit, whereas several flours exceeded it. This variation suggests that maintaining uniform alkaloid reduction remains an important quality control objective for the food industry to ensure high consumer safety standards.

### 3.2. Profile and Disribution of Predominant Alkaloids in Analyzed Samples

All quinolizidine alkaloids identified in lupin species originate from the precursor amino acid L-lysine, which is metabolized into primary core structures, including sparteine and lupanine. Characterized by its central role in the biosynthetic pathway, lupanine acts as the primary metabolic precursor for the synthesis of other QAs [3,8].

Of the samples analyzed, the predominant QA was lupanine, with the exception of lupin flours, where 13α-hydroxylupanine prevailed (Table 2). Notably, albine and 13α-hydroxylupanine were ubiquitously detected across almost all samples, exhibiting significantly higher concentrations in dried lupins compared to lupin flours (Table 3).

The alkaloids cytisine, N-methylcytisine, thermopsine and 13α-cinnamoyloxylupanine were below the LOQ. Anagyrine was identified in only one sample of dried lupin seeds, whereas gramine, a non-quinolizidine indole alkaloid typically found in yellow lupin (*L. luteus*) [3], was detected in four samples of lupin flour. Similarly, lupinine, another characteristic alkaloid of *L. luteus*, was not detected in any of the analyzed dried lupin samples, but was found exclusively in lupin flours and in one ready-to-use product.

Literature data indicate that pure lines of *L. albus* exhibit a characteristic quinolizidine alkaloid profile dominated by lupanine (up to 88–97%) [13,25,26], followed by significant proportions of albine (up to 26%), 13α-hydroxylupanine (up to 24%), and multiflorine (up to 11%), as detailed in Table 4. This specific alkaloid distribution represents a distinctive trait of white lupin, differentiating it from other widely cultivated food-grade species such as *Lupinus angustifolius*, *Lupinus luteus*, and *Lupinus mutabilis*, each characterized by its own unique major and secondary QA markers.

Consistent with these literature findings, the baseline profile of the analyzed whole-lupin seeds closely matched the typical *L. albus* fingerprint. As reported in Table 5, dried lupins exhibited a median distribution of 71% lupanine, 11% albine, 9% 13α-hydroxylupanine, and 6% multiflorine, while brined seeds showed a closely aligned pattern, consisting of 67% lupanine, 16% albine, 8% 13α-hydroxylupanine, and 8% multiflorine.

Given that albine is an exclusive botanical marker for *L. albus*, its constant identification confirms that white lupin constitutes the primary species for the commercial products surveyed across the Italian retail market. However, while dried and brined seeds maintain this stable, lupanine-dominated phenotypic profile, the processed matrices, particularly lupin flours, reveal a significant compositional shift, characterized by a decrease in lupanine to 37% and a concomitant increase in 13α-hydroxylupanine, its metabolite, to 32%. This transition from a mono-dominant to a more balanced profile in processed products likely does not indicate a change in botanical origin but rather a combination of technological and biological drivers occurring during industrial manufacturing. On one hand, this shift reflects the differential structural stability of individual QAs during mechanical and thermal processing.

As documented by [25,27], closed multi-ring molecular structures, such as lupanine and 13α-hydroxylupanine, exhibit high resistance to dry heat and washing compared to open-ring structures like albine. Consequently, industrial thermal and mechanical treatments (e.g., milling or roasting) alter the percentage ratios due to differentiated degradation kinetics.

On the other hand, the bio-metabolic activity of the seeds during initial conditioning and hydration must be considered. In these early phases, the seeds are still alive and undergo germination processes, mobilizing nitrogen from alkaloid reserves to support protein synthesis stimulating endogenous hydroxylases, driving the biological bioconversion of lupanine into 13α-hydroxylupanine before the final thermal stabilization and milling steps fix the profile [28,29].

Indeed, according to [30], sweet lupin varieties naturally display a balanced alkaloid distribution, whereas bitter cultivars are typically dominated by lupanine. Therefore, this balanced profile, compared to the bitter, can result from both the genetic trait of sweet cultivars and the combined effects of early metabolism and industrial processing. Consequently, alkaloid profiling via LC-MS/MS remains effective for confirming the species profile in whole seeds but becomes insufficient for processed products. In these commercial forms, genetic analysis is required to definitively verify the botanical origin.

### 3.3. Efficiency of the Debittering Process

To evaluate QA reduction kinetics, the eight dried lupin samples (DL 1–DL 8) were subjected to a laboratory-scale debittering process (Table 6). The treatment achieved a substantial reduction percentage (R%) ranging from 94% to 99%, in line with the EFSA scientific opinion [8]. However, the process efficiency often did not correlate with the initial alkaloid content. For instance, sample DL 6, which started with the highest concentration (18,662.6 mg/kg), achieved the highest reduction (99%), leaving a residual concentration of 207.0 mg/kg. Conversely, a lower removal efficiency was noted in sample DL 1; despite having the lowest starting level (7716.0 mg/kg), it retained a remarkably high residual content of 470.4 mg/kg. Crucially, despite the high R% (94–99%), residual QA levels in all eight debittered samples remained above the 200 mg/kg (FSANZ safety limit). This finding supports the public health concerns raised by other scientists regarding domestic preparation [7]. Household preparation protocols are inherently non-standardized, and the generic, incomplete instructions currently provided on retail labels fail to guarantee consumer safety when highly contaminated batches enter the market.

To effectively mitigate this toxicological risk, interventions are required on two levels: first, at the market supply chain level, by commercializing selected low-alkaloid “sweet” lupin varieties for consumer packaging; second, at the labeling level, by enforcing validated and standardized debittering protocols directly on retail packages.

## 4. Conclusions

This study provides a comprehensive overview of the occurrence and distribution of quinolizidine alkaloids across the Italian retail lupin market using a validated LC-MS/MS methodology. Commercial finished products, such as lupin flours, brined seeds, and ready-to-use alternatives, generally exhibit lower QA concentrations due to industrial selection and processing, while dried seeds pose a major risk prior to the debittering process.

Furthermore, the laboratory debittering simulations demonstrated that despite achieving remarkably high removal efficiencies (R% up to 99%), traditional household protocols may not consistently lower QA concentrations to negligible amounts. Our findings highlight that when dried seeds carry an elevated initial QA load, substantial alkaloid content can still persist in the final product, exceeding the FSANZ reference value.

While these higher residual concentrations suggest the need for consumer caution, a definitive estimation of the actual consumer health risk cannot be postulated based on these data alone, as it would require a comprehensive dietary exposure assessment.

To mitigate this, preventive strategies must be implemented directly in the field. This requires the selection of sweet lupin cultivars coupled with continuous pre-harvest monitoring, as climate-induced crop stress can trigger a defensive increase in QA production.

As the market for sustainable, plant-based protein sources continues to expand within Europe, these results highlight the clear benefit and importance of establishing harmonized maximum regulatory QA levels within the European Union.

To support this normative framework, future research will focus on monitoring a broader sample size to systematically assess QA levels across both processed matrices and dried seeds. This monitoring aims to provide robust data for a comprehensive population exposure assessment, particularly evaluating household debittering practices performed by consumers according to the instructions provided on manufacturer labels.

Therefore, continuous analytical market surveillance, the integration of pre-harvest quality controls, and the enforcement of safety limits represent essential requirements to guarantee consumer safety.

## Figures and Tables

**Table 1:** Lupin samples analyzed.

Food Category	Number of Samples
Lupine flour	8
Brined lupins	10
Dried lupins	8
Ready-to-use lupin-based products ^1^	2

^1^ Vegetarian salami and lupin snack.

**Table 2:** QA levels in analyzed samples (mg/kg).

Food Category	QA Mean	QA Min	QA Max	QA Median	Predominant QA
Lupin flour	253.0	7.7	721.0	268.6	13α-hydroxylupanine
Brined lupins	86.1	12.7	320.8	51.2	Lupanine
Dried lupins	13,676.0	7716.0	18,662.6	14,176.7	Lupanine
Ready-to-use lupin-based products	70.3	60.7	80.0	70.3	Lupanine

**Table 3:** Quinolizidine alkaloid profile in lupin products and corresponding food categories with the highest concentrations (mg/kg).

Quinolizidine Alkaloids	Detected Range	Mean Value	Median Value	Food Categories ^2^
Lupanine	3.0–13,478.0	2813.8	92.3	Dried lupins; brined lupins
Albine	0.9–2104.0	448.0	34.1	Dried lupins; lupin flour
13α-hydroxylupanine	1–1483.0	413.1	77.5	Dried lupins; lupin flour
Angustifoline	0.9–420.1	120.0	25.7	Dried lupins; lupin flour
Lupinine	0.2–2.9	0.6	0.3	Ready-to-use product
Multiflorine	0.2–1080.0	261.6	6.6	Dried lupins; lupin flour
Sparteine	0.3–42.8	18.9	19.3	Dried lupins; brined lupins
Cytisine	<LOQ ^1^	<LOQ	<LOQ	<LOQ
N-methylcytisine	<LOQ	<LOQ	<LOQ	<LOQ
Anagyrine	2.8	<LOQ	<LOQ	Dried lupins
Thermopsine	<LOQ	<LOQ	<LOQ	<LOQ
13α-cinnamoyloxylupanine	<LOQ	<LOQ	<LOQ	<LOQ
Gramine	0.2–0.6	0.3	0.3	Lupin flour
QA sum	7.7–18,662.6	3888.4	241.4	Dried lupins; lupin flour

^1^ LOQ lupin seeds = 1 mg/kg and LOQ lupin-based products = 0.2 mg/kg; ^2^ food categories with the highest individual alkaloid content among those analyzed.

**Table 4:** Typical QA profile of the major cultivated lupin species used for food products, adapted from [8,13].

Names	White Lupin	Narrow-Leaved Lupin	Yellow Lupin	Andean Lupin
**Species**	*Lupinus albus*	*Lupinus angustifolius*	*Lupinus luteus*	*Lupinus mutabilis*
I QAs ^1^	Lupanine (up to 97%)	Lupanine (up to 82%)	Sparteine (up to 97%)	Lupanine (up to 88%)
	Albine (up to 26%)	13α-Hydroxylupanine (up to 47%)	Lupinine (up to 46%)	Sparteine (up to 23%)
	13α-hydroxylupanine (up to 24%)	Angustifoline (up to 31%)		13α-hydroxylupanine (up to 15%)
	Multiflorine (up to 11%)	Isolupanine (up to 14%)		
II QAs ^2^	13α-Angeloyloxylupanine ^3^	Sparteine	13α-hydroxylupanine	Tetrahydrorhombifoline
	Angustifoline	Tetrahydrorhombifoline ^4^	Lupanine	13α-angeloyloxylupanine
	Isolupanine	Multiflorine	Gramine	
	Sparteine			

^1^ I QAs = predominant QAs; ^2^ II QAs = secondary QAs; ^3,4^ molecules not investigated in this study.

**Table 5:** Median percentage distribution of predominant quinolizidine alkaloids across the analyzed food categories.

Food Category	Quinolizidine Alkaloids ^1^			
	Lupanine % ^2^	Albine %	13α-Hydroxylupanine %	Multiflorine %
Dried lupins	71	11	9	6
Brined lupins	67	16	8	8
Lupin flour	37	15	32	1
Ready-to-use Products	50	21	14	7

^1^ Values indicate only the QAs generally predominant in *L. albus*; ^2^ median values %.

**Table 6:** Results of debittered samples and QA reduction percentage (R%), expressed in mg/kg.

Dried Lupin Samples	Dried QAs	Debittered QAs ^1^	R%
DL 1	7716.0	470.4	94%
DL 2	10,895.0	335.4	97%
DL 3	13,223.7	578.2	96%
DL 4	15,732.8	546.0	97%
DL 5	14,830.2	460.3	97%
DL 6	18,662.6	207.0	99%
DL 7	13,964.3	530.1	96%
DL 8	14,389.2	548.3	96%

^1^ Ready-to-eat seeds.

## Data Availability

The original contributions presented in the study are included in the article/Supplementary Materials; further inquiries can be directed to the corresponding author.

## Supplementary Materials

The following supporting information can be downloaded at https://www.mdpi.com/article/10.3390/foods15132269/s1, Table S1: UPLC-MS/MS system conditions; Table S2: LC-MS/MS parameters for all quinolizidine alkaloids (CE: collision energy, Q: quantifier ion, q: qualifier ion); Table S3: Concentration of individual quinolizidine alkaloids detected and their sum in all analyzed samples.
