# Supplementary material for: Quinolizidine Alkaloid Profiles in Lupin-Based Products: Monitoring of the Italian Retail Market and Efficacy of the Debittering Process
Source: Foods. 2026 Jun 24;15(13):2269. doi: 10.3390/foods15132269 (PMC13361213; doi:10.3390/foods15132269)
Supplement: Supplementary file 1 [file foods-15-02269-s001.zip › foods-4377021-supplementary.pdf]

foods

Supplementary Materials

Quinolizidine Alkaloid Profiles in Lupin-Based Products: Monitoring of the Italian Retail Market and Efficacy of the Debittering Process

Mariantonietta Peloso, Ilaria Prizio, Gaetan Minkoumba Sonfack, Eleonora Baraldini Molgora and Elisabetta Caprai *

National Reference Laboratory for Plant Toxins in Food, Food Chemical Department, Istituto Zooprofilattico Sperimentale della Lombardia e dell'Emilia-Romagna "Bruno Ubertini" (IZSLER), Via P. Fiorini 5, 40127 Bologna, Italy; m.peloso@izsler.it (M.P.); ilaria.prizio@izsler.it (I.P.); g.minkoumbasonfack@izsler.it (G.M.S.); e.baraldinimolgora@izsler.it (E.B.M.)

* Correspondence: elisabetta.caprai@izsler.it

1. Tables Content—Materials and Methods

Table S1. UPLC-MS/MS system conditions

System and Parameters	UPLC conditions
LC-MS/MS equipment	XEVO TQ-Xs Acquity UPLC I Class Plus Waters
LC Column	UPLC BEH C18 Waters
Mobile Phase A	5 mM ammonium formate and 0.1% formic acid in water
Mobile Phase B	5 mM ammonium formate and 0.1% formic acid in methanol
Flow (mL/min)	0.40
Injection volume (μL)	1
Ionization mode	ESI+
Capillary (kV)	1.40
Cone voltage (V)	24
Source Temperature (◦C)	600
Desolvation Gas Flow (L/h)	1000
Cone Gas Flow (L/h)	150

Table S2. LC-MS/MS parameters for all Quinolizidine Alkaloids (CE: collision energy, Q: quantifier ion, q: qualifier ion)

Quinolizidine Alkaloids	MH+	CE	m/z	Q, q
Albine	233.2	20	112.0	Q
		20	138.0	q
Angustifoline	235.2	20	112.0	Q
		30	193.0	q
Lupanine	249.2	20	136.0	Q
		25	114.0	q
Lupinine	170.2	20	152.0	Q
		25	124.0	q
Cytisine	191.2	20	148.0	Q
		30	133.0	q
N-methylcytisine	205.2	20	108.0	Q

		20	160.0	q
Gramine	130.0	20	77.0	Q
		25	103.0	q
Multiflorine	247.2	25	112.0	Q
		40	70.0	q
Thermopsine	245.2	35	98.0	Q
		45	70.0	q
Sparteine	235.2	30	98.0	Q
		30	233.0	q
Trans-13α- cinnamoyloxylupanine	395.2	30	247.0	Q
		30	112	q
Isolupanine	249.2	30	98.0	Q
		30	84.0	q
Anagyrine	245.2	35	98.0	Q
		45	70.0	q
13α-hydroxylupanine	265.2	30	152.1	Q
		28	114.1	q

2. Tables Content—Results

Table S3. Concentration of individual Quinolizidine Alkaloids detected and their sum in all analyzed samples

Sample number	Food Categories	Quinolizidine Alkaloids [1] (mg/kg)										Sum of QAs
		Albine	Anagyrine	Angustifoline	Gramine	13α-hidroxylupanine	Isolupanine	Lupanine	Lupinine	Multiflorine	Sparteine	
1	Brined Lupins	3.4	<LOQ [2]	1.3	<LOQ	2.1	<LOQ	16.0	<LOQ	2.7	<LOQ	25.5
2	Brined Lupins	37.4	<LOQ	9.0	<LOQ	19.4	2.3	221.4	<LOQ	27.2	4.1	320.8
3	Brined Lupins	6.0	<LOQ	<LOQ	<LOQ	<LOQ	<LOQ	3.4	<LOQ	3.3	<LOQ	12.7
4	Brined Lupins	4.8	<LOQ	<LOQ	<LOQ	1.8	<LOQ	16.4	<LOQ	2.1	<LOQ	25.1
5	Brined Lupins	2.2	<LOQ	<LOQ	<LOQ	1.0	<LOQ	9.5	<LOQ	1.0	<LOQ	13.7
6	Brined Lupins	<LOQ	<LOQ	<LOQ	<LOQ	29.1	7.6	90.6	<LOQ	<LOQ	<LOQ	127.3
7	Brined Lupins	8.9	<LOQ	1.8	<LOQ	3.5	<LOQ	32.3	<LOQ	7.4	<LOQ	53.9
8	Brined Lupins	7.3	<LOQ	2.6	<LOQ	6.1	<LOQ	52.2	<LOQ	3.3	<LOQ	71.5
9	Brined Lupins	37.3	<LOQ	6.0	<LOQ	14.5	<LOQ	83.7	<LOQ	18.8	2.2	162.5
10	Brined Lupins	5.3	<LOQ	1.6	<LOQ	4.2	<LOQ	35.1	<LOQ	2.3	<LOQ	48.5
11	Dried Lupins	691.0	<LOQ	182.0	<LOQ	892.0	44.0	5,445.0	<LOQ	446.0	16.0	7,716.0
12	Dried Lupins	966.0	<LOQ	259.0	<LOQ	1,252.0	69.0	7,706.0	<LOQ	615.0	28.0	10,895.0

13	Dried Lupins	1,473.6	<LOQ	327.6	<LOQ	1,258.6	87.7	9,324.7	<LOQ	718.2	33.3	13,223.7
14	Dried Lupins	1,710.8	<LOQ	392.6	<LOQ	1,483.7	96.6	10,955.2	<LOQ	1,051.1	42.8	15,732.8
15	Dried Lupins	1,800.0	<LOQ	349.3	<LOQ	1,357.6	96.7	10,252.7	<LOQ	941.7	32.2	14,830.2
16	Dried Lupins	2,174	2.8	420.1	<LOQ	1,368.6	107	13,478.5	<LOQ	1,080.8	30.8	18,662.6
17	Dried Lupins	1,487.9	<LOQ	340.2	<LOQ	1,330.1	72.9	9,874.3	<LOQ	824.2	34.7	13,964.3
18	Dried Lupins	1,385.3	<LOQ	315.8	<LOQ	1,315.6	76.9	10,305.8	<LOQ	970.5	19.3	14,389.2
19	Lupin Flour	50.9	<LOQ	27.5	0.2	107.2	15.9	114.5	0.4	4.1		320.7
20	Lupin Flour	1.0	<LOQ	113.6	0.6	290.3	59.1	198.9	<LOQ	56.1	2.0	721.6
21	Lupin Flour	33.5	<LOQ	23.7	<LOQ	80.8	11.2	93.9	0.2	2.9	<LOQ	246.2
22	Lupin Flour	0.9	<LOQ	0.9	<LOQ	2.5	0.2	3.0	<LOQ	0.2	<LOQ	7.7
23	Lupin Flour	34.1	<LOQ	24.8	<LOQ	77.5	11.3	90.5	0.2	3.0	<LOQ	241.4
24	Lupin Flour	25.4	<LOQ	18.1	<LOQ	64.1	9.2	76.5	0.2	2.5	<LOQ	196.0
25	Lupin Flour	46.2	<LOQ	29.1	0.2	91.0	13.8	106.6	0.3	3.8	<LOQ	291.0
26	Lupin Flour	72.2	<LOQ	26.5	0.3	79.2	10.6	129.1	0.3	5.8	0.3	324.3
27	Ready-to-use product	16.1	<LOQ	3.7	<LOQ	16.6	3.3	37.4	2.9	<LOQ	<LOQ	80.0
28	Ready-to-use product	13.3	<LOQ	2.2	<LOQ	3.9	0.3	32.3	<LOQ	8.2	0.5	60.7

[1] Cytisine, N-methylcytisine, Thermopsine and 13α-cinnamoyloxylupanine are not included in the Table as their concentrations were below the LOQ in all analyzed samples; [2] LOQ lupin seeds=1 mg/kg and LOQ lupin-based products= 0.2 mg/kg.
